# Top-down matching singleton cues have no edge over top-down matching nonsingletons in spatial cueing

**DOI:** 10.3758/s13423-018-1499-5

**Published:** 2018-06-29

**Authors:** Tobias Schoeberl, Florian Goller, Ulrich Ansorge

**Affiliations:** Faculty of Psychology, University of Vienna, Liebiggasse 5, 1010 Wien, Austria

**Keywords:** Attention, Contingent capture, Stimulus-driven capture

## Abstract

In the present study, we investigated in a novel version of the peripheral-cueing paradigm whether object salience influences attentional selection at early stages of visual processing. In each trial, participants searched for targets of one of two possible colors. In the most important condition, the cueing displays consisted of a singleton cue having one target color and three additional nonsingletons of another target color. Hence, all objects in these all-relevant cueing displays had a target color. If singletons initially capture attention in a stimulus-driven way, regular cueing effects (faster responses to targets at the cued location than to targets away from the cue) should be found in these conditions. However, the results suggested otherwise: As compared to a control condition with a singleton cue of a target color among nonsingletons of a nontarget color, the cueing effects in all-relevant cueing displays were strongly reduced. This was also replicated with a very brief cue–target interval. The results suggest top-down contingent capture of attention even during the initial phase of processing salient stimuli, and argue against stimulus-driven capture of attention plus subsequent rapid disengagement.

Bottom-up theories of attention capture claim that salient objects capture attention automatically in a stimulus-driven way (e.g., Theeuwes, 1992, 2010). According to this view, feature singletons, items that pop out by a particular feature relative to the surrounding objects, capture attention independently of the current search goals of the observer. In contrast, proponents of top-down models argue that attentional capture is contingent on top-down search settings of the observer (e.g., Folk, Remington, & Johnston, 1992), that is, even highly salient feature singletons would only capture attention when they match the observer’s search settings.

A typical pattern of results that speaks to the latter possibility and against bottom-up theories is the *contingent-capture effect* (Folk et al., 1992): When participants search for a predefined feature target, an unpredictive peripheral cue with the searched-for target feature (matching cue) that is presented prior to the target leads to *cueing effects*: faster responses to targets at cued locations than at locations away from the cue. For instance, presenting a red cue prior to a target that is defined by its red color facilitates search if the cue and target are presented at the same position (in the valid condition), relative to their presentation at different positions (in the invalid condition). This cueing effect reflects attentional capture by the cue.^1^ Importantly, cueing effects are found only with matching cues. Cues without the target’s searched-for features (nonmatching cues) do not lead to cueing effects. This pattern of results has been interpreted as evidence that attentional capture is contingent on top-down search settings, because top-down nonmatching cues do not lead to cueing effects, even when they are presented as salient feature singletons (Folk et al., 1992).

Bottom-up theories have proposed ways to accommodate the lack of cueing effects with nonmatching cues. For example, according to the *rapid-disengagement hypothesis* (Belopolsky, Schreij, & Theeuwes, 2010; Theeuwes, 2010; Theeuwes, Atchley, & Kramer, 2000), salient yet nonmatching cues do capture attention in a bottom-up way. However, no overt cueing effects are found because the focus of attention rapidly disengages from the cue’s location. Central to this idea is that the speed of disengagement depends on the presence of a match between the features of the cue and those of the searched-for target. When the features of the cue match the searched-for target features, attention dwells at the corresponding location, and hence, cueing effects are found. Only when the features of the cues mismatch the target features can participants rapidly withdraw attention from the cue’s location, so that no overt cueing effect is found.

Rapid disengagement in its original form has sometimes been doubted (e.g., Ansorge, Kiss, Worschech, & Eimer, 2011; Gaspelin, Leonard, & Luck, 2015; Sawaki & Luck, 2010). However, the strength of initial salience-based bottom-up capture is still a matter of debate (cf. Gaspelin et al., 2015; Sawaki & Luck, 2010; see also DiQuattro & Geng, 2017; Fukuda & Vogel, 2011).

Here we took a novel approach to study the strength of bottom-up capture. The major objective was to test to what extent feature singletons would capture attention when they were top-down matching but were presented among nonsingletons that were *also* top-down matching. If attentional capture only depended on a match between the object features and top-down search settings, we would expect more salient items not to have an edge over less salient items when all items in the display have task-relevant features. In contrast, we would expect to see attentional capture by the most salient item if bottom-up models are true (especially if the rapid-disengagement hypothesis holds). The reason is that the most salient items in the display would capture attention automatically, and disengagement from or suppression of salient items would be ruled out because the features of the corresponding object would match the search settings.

To test this, we employed a novel *all-relevant cueing display* in a spatial-cueing protocol: In each trial, participants searched for two target colors (e.g., red and blue).^2^ One of these relevant colors was used for the salient top-down-matching singleton cue, and the other one of these relevant colors was used for the nonsingleton cueing-display distractors. This allowed us to create cueing displays of minimal disengagement from the singleton cue, because all items (both the cue and the cueing-display distractors) matched the top-down search setting. Yet just one of these items—the cue—was a singleton.^3^ As we explained above, we would expect to find more attentional capture by the singleton cues in all-relevant cueing displays if bottom-up theories are true. In contrast, we would expect to find no more capture of attention by singleton cues than by all other objects in the all-relevant cueing displays if top-down models are true.

We also replicated the standard cueing effect with a top-down-matching singleton cue among nonmatching nonsingletons. We refer to this condition as the *standard matching condition*. In addition, we also included cueing displays in which neither the singleton cue nor the nonsingletons had a searched-for target feature, and thus they were nonmatching. This was the *standard nonmatching condition*. Finally, in one condition only the nonsingletons in the cueing display had a target color, but the singleton cue did not. This was the *nonsingleton relevant condition*.

We also investigated the influence of intertrial priming on attention capture (e.g., Folk & Remington, 2008): We tested to what extent cueing effects depended on the similarity between the color of the target in a preceding trial *n*−1 and the color of the cue in a given trial *n*. Under the priming hypothesis, cueing effects should be larger when the target color in *n*−1 is similar to the cue’s color in trial *n* (Theeuwes, 2013). We did this to test whether the residual cueing effects in the all-relevant conditions were due to priming.

## Experiment 1

### Method

#### Participants

Eighteen participants took part (14 female, four male; *M*_age_ = 23.17 years, *SD*_age_ = 2.04 years). Two of the participants were excluded because their error rates exceeded 40%.

#### Apparatus and stimuli

**Fig. 1:**
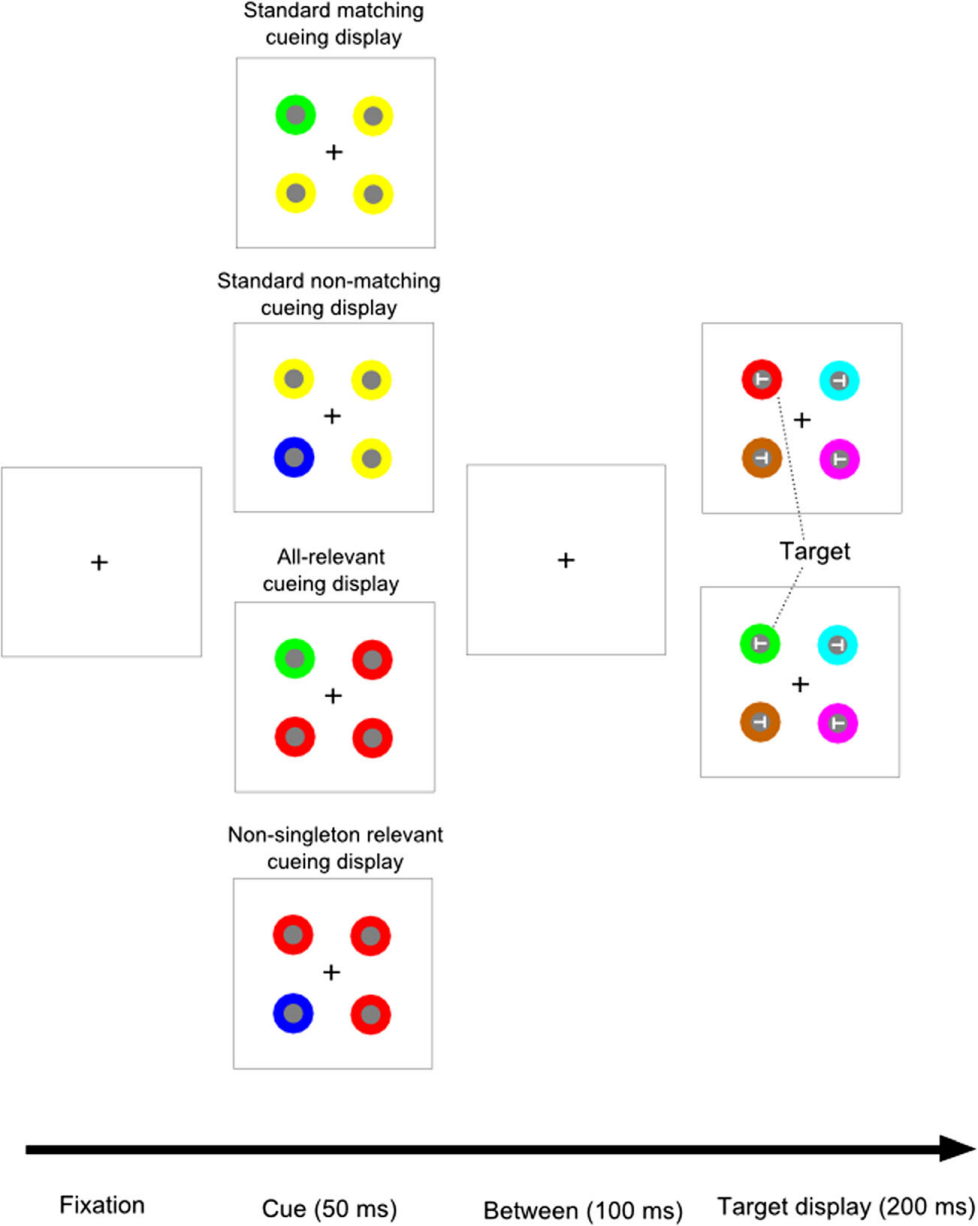
Schematic sequence of events in Experiment 1 with the different cueing displays. See the online publication for the color version of the figure. The arrow at the bottom depicts the flow of time, from left to right. In the depicted case, participants would be instructed to search for a target in either green or red

The stimuli were presented against a gray background (CIELAB color coordinates: 55.0, − 2.3, − 14.4) on an LCD monitor, with a refresh rate of 60 Hz and a resolution of 1,280 × 1,024 pixels. The stimuli, centered 4.9° of visual angle from a fixation cross (~ 0.5°) at screen center, were dark gray circles (1° diameter; CIELAB: 35.2, 2.4, − 8.7) surrounded by 0.5°-thick colored rings in yellow (CIELAB: 56.5, − 17.2, 44.8), green (53.2, − 59.0, 34.0), red (56.8, 72, 50.4), blue (52.2, 75.5, − 156.9), turquoise (55.4, − 33.2, − 24.2), pink (56.9, 80.8, − 68.3), or brown (57.5, 17.4, 52.1). The cueing and target displays consisted of four stimuli at equidistant locations (see Fig. 1). Only in the target display were two leftward- and two rightward-tilted Ts (width and height: 0.5°) shown, in light gray (CIELAB: 78.9, − 4.0, − 19.5), one centered on each disk (cf. Carmel & Lamy, 2014).

#### Design

Participants performed 1,152 trials in nine blocks of 128 trials each. After each block, there was a break with feedback about a participant’s percent correct responses up to this point. Participants were instructed to search for a target that could appear in two possible colors and to discriminate the orientation of the T inside of the target. They had to press the “J” key on a standard keyboard for a rightward- and the “F” key for a leftward-tilted Ts.

Each trial began with a fixation cross. The cueing display was presented after 1 s, with one of four items as a singleton cue of a unique color relative to the three nonsingletons. The cue was either in one of the target colors (matching cue) or in a nontarget color (nonmatching cue). The colors of targets and cues were chosen from the three colors red, green, and blue. (Which two colors were used for the targets and matching cue and which was used for the nonmatching cue was balanced across participants.) The nonsingletons in the cueing display had either a nontarget color or a target color different from that of the cue color.

The cueing display of 50 ms was followed by a blank interval with only the fixation cross for 100 ms. Next, the target display was presented for 200 ms. It consisted of one target and three differently colored distractors—that is, each item had its own color, to prevent singleton search for targets (Bacon & Egeth, 1994). Participants discriminated the orientation of the target T. If participants failed to respond or responded incorrectly, the trial counted as an error. Trial-wise feedback was given only at the beginning, during practice. The experiment started when the participants got at least 70% correct in 20 practice trials.

In each trial, the target and cue appeared randomly and unpredictably at one of four possible locations. Steps of the variables cue location, target location, and cue color (target color vs. nontarget color) were combined orthogonally. On each trial, the trial type was selected pseudorandomly.

### Results

#### Reaction times

**Fig. 2:**
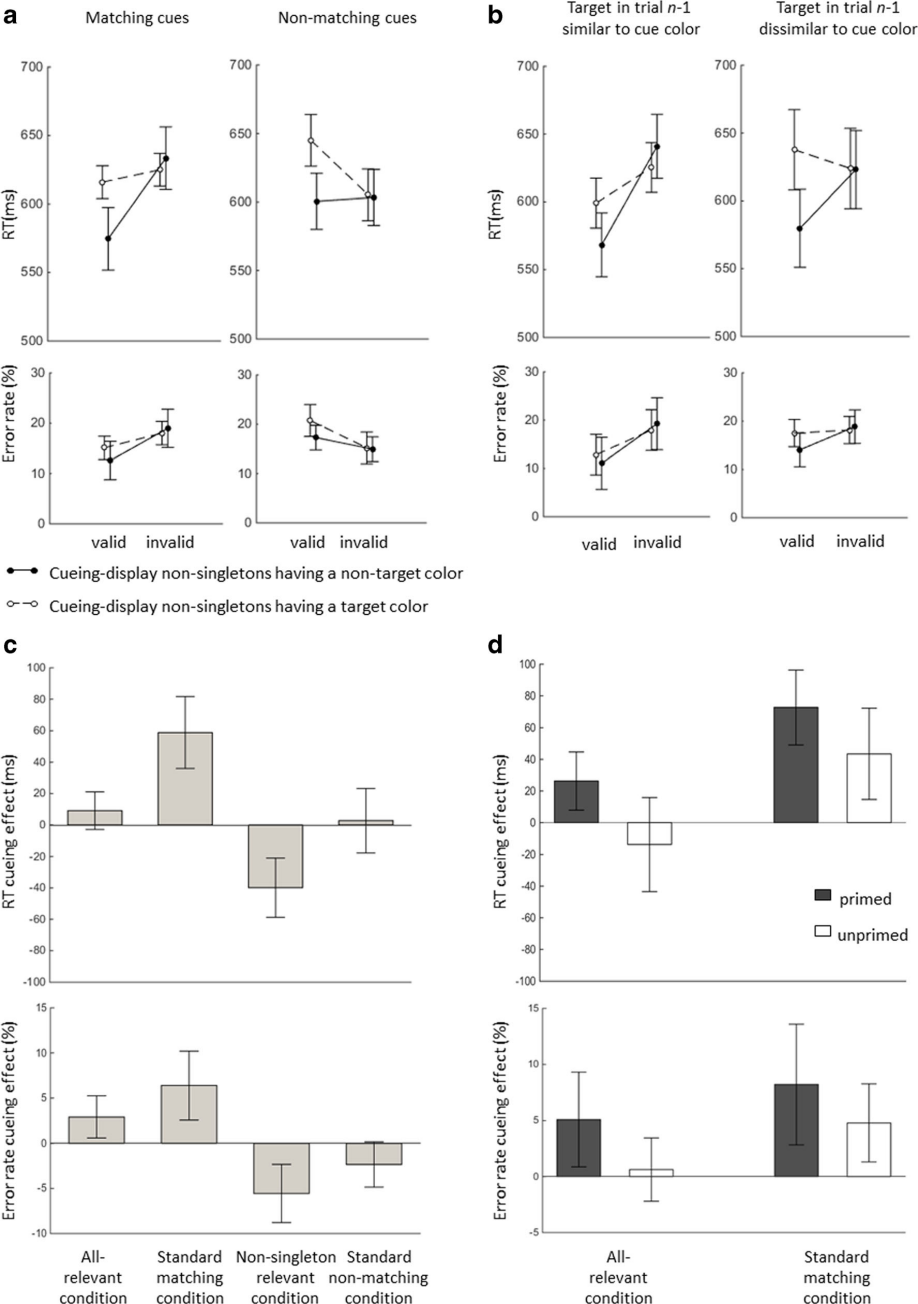
Data from Experiment 1. (A) Overall means of the reaction times (RTs) and error rates for the top-down matching and nonmatching cues. (B) Mean RTs and error rates for top-down-matching cues when the target in trial *n*−1 was similar to the cue in trial *n* (i.e., when the cue was primed) and when the color of the target in trial *n*−1 was dissimilar to the cue color in trial *n* (i.e., when the cue was not primed). Error bars represent the 95% confidence interval (CIs) of the invalid minus valid differences in the corresponding conditions. (C) Mean cueing effects (difference between mean RTs and error rates in the invalid and valid conditions) with different cueing displays. The upper panel corresponds to the cueing effects on RTs, and the lower panel corresponds to the cueing effects on error rates. The error bars depict the 95% CIs. (D) Mean cueing effects and CIs for RTs and error rates in the standard matching cue condition and in the all-relevant condition for trials in which the cue colors were primed and unprimed by the target color in trial *n*−1

Trials deviating by more than 2.5 *SD*s from the mean (2.0% of trials) were excluded. The data are shown in Fig. 2. The trimmed correct mean reaction times (RTs) were subjected to a repeated measurements analysis of variance (ANOVA) with the variables validity (valid vs. invalid), cue match (top-down matching vs. nonmatching), and nonsingleton color (target color vs. nontarget color; i.e., whether the nonsingletons in the cueing display had a target color). Table 1 shows the results. Most importantly, we observed two-way interactions between validity and cue match, *F*(1, 15) = 20.73, *p* < .001, *η*_p_^2^ = .58, and between validity and nonsingleton color, *F*(1, 15) = 26.57, *p* < .001, *η*_p_^2^ = .64.

**Table 1:** ANOVA results for Experiment 1, with the variables validity, cue match, and nonsingleton color

Source	*df*	*df* (error)	ANOVA on Reaction Times			ANOVA on Error Rates		
			*F*	*p*	*η*_p_^2^	*F*	*p*	*η*_p_^2^
Validity	1	15	4.52	.05	.23	0.49	.50	.03
Cue match	1	15	0.23	.64	.02	1.37	.26	.08
Nonsingleton color	1	15	41.15	<.01^**^	.73	1.44	.25	.09
Validity × Cue match	1	15	20.73	<.01^**^	.58	32.51	<.01^**^	.68
Validity × Nonsingleton color	1	15	26.57	<.01^**^	.64	4.32	.06	.22
Cue match × Nonsingleton color	1	15	1.41	.25	.09	1.87	.19	.11
Validity × Cue match × Nonsingleton color	1	15	0.25	.63	.02	0.01	.93	.00

^**^p < .01

To understand the interactions, cueing effects (invalid RT minus valid RT) were tested against zero. We also obtained Bayes factors (BF_10_) from Bayesian *t* tests using the JASP software (Marsman & Wagenmakers, 2016). There was only a nonsignificant trend toward a cueing effect with all-relevant cueing displays, 9 ms, *t*(15) = 1.62, one-tailed *p* = .06, one-sided BF_10_ = 1.39. In the standard matching cue conditions, we found a regular cueing effect, 59 ms, *t*(15) = 5.49, *p* < .001, BF_10_ = 431.93. With the nonsingleton relevant cueing displays (in which the cues were nonmatching and only the distractors had a target-matching color), the cueing effect reversed, − 40 ms, *t*(15) = 4.50, *p <* .001, BF_10_ = 80.69. There was no cueing effect in the standard nonmatching condition, 3 ms, *t*(15) = 0.29, *p* = .78, BF_10_ = 0.27.

#### Error rates

The results of an ANOVA on the percentages of errors per each condition can also be found in Table 1. An interaction emerged between validity and cue match, *F*(1, 15) = 32.51, *p* < .001, *η*_p_^2^ = .68, and a trend toward an interaction between validity and nonsingleton color, *F*(1, 15) = 4.32, *p* = .055, *η*_p_^2^ = .22.

Follow-up *t* tests revealed a small cueing effect, 3%, *t*(15) = 2.68, *p* < .05, BF_10_ = 3.50, for all-relevant cueing displays. The cueing effect was also present in standard matching cue conditions, 6%, *t*(15) = 3.58, *p* < .01, BF_10_ = 16.28. This effect was again reversed with nonsingleton relevant cueing displays, − 6%, *t*(15) = 3.67, *p* < .01, BF_10_ = 19.07. In the standard nonmatching cue conditions, there was a trend toward a reversed cueing effect, − 2%, *t*(15) = 1.99, *p =* .07, BF_10_ = 1.23.

### Intertrial priming

We investigated intertrial priming of capture by computing the mean RTs as described above, once in conditions with targets in trial *n*−1 that preceded matching cues of the same color in trial *n*, and once with targets that preceded matching cues of a different color. Table 2 shows the results of the ANOVA, with the variables validity (valid vs. invalid), target *n*−1 (similar vs. dissimilar to cue in *n*), and nonsingleton color (target color vs. nontarget color). The most important result of the intertrial analysis in the present context was that in unprimed all-relevant cueing displays (i.e., when the current cue had had a color different from the target in trial *n*−1), the cueing effect was numerically reversed, − 14 ms, *t*(15) = 0.99, *p* = .34, BF_10_ = 0.39. This shows that, other than priming of capture, there was no cueing effect with all-relevant cueing displays. Reliable cueing effects were not observed, either, for the error rates in the all-relevant conditions when the cue was not primed, 0.6%, *t*(15) = 0.47, *p* = .65, BF_10_ = 0.28. This is at odds with the bottom-up singleton capture view. At the same time, the RT cueing effect was present in unprimed control conditions with standard matching cues, 43 ms, *t*(15) = 3.22, *p* < .01, BF_10_ = 8.63. This supported our assumption that the matching cue was in principle able to capture attention; it only failed to do so when competing with other matching stimuli in the all-relevant conditions.

**Table 2:** Results of ANOVAs of intertrial priming effects in Experiment 1, with the variables validity, target *n*−1, and nonsingleton color

Source	*df*	*df* (error)	ANOVA on Reaction Times			ANOVA on Error Rates		
			*F*	*p*	*η*_p_^2^	*F*	*p*	*η*_p_^2^
Validity	1	15	30.58	<.01^**^	.67	17.29	<.01^**^	.54
Target *n*−1	1	15	3.57	.08	.19	4.78	<.05^*^	.24
Nonsingleton color	1	15	22.79	<.01^**^	.60	0.63	.44	.04
Validity × Target *n*−1	1	15	6.62	.02^*^	.31	3.16	.10	.17
Validity × Nonsingleton color	1	15	14.39	<.01^**^	.49	3.79	.07	.20
Target *n*−1 × Nonsingleton color	1	15	3.42	.08	.19	0.69	.42	.04
Validity × Target *n*−1 × Nonsingleton color	1	15	0.43	.52	.03	0.22	.65	.01

^*^p < .05, ^**^p < .01

## Experiment 2

We reduced the stimulus onset asynchrony (SOA) between cue and target to 50 ms, to see whether more evidence of capture by top-down matching singleton cues in all-relevant cueing displays could be observed with less time for deallocation (cf. Kim & Cave, 1999; see also Chen & Mordkoff, 2007).

### Method

Eighteen new participants took part (ten female, eight male; *M*_age_ = 22.28 years, *SD*_age_ = 3.46). Experiment 2 was similar to Experiment 1, except for the SOA: Now the blank display between the cueing and target displays was left out, so that the SOA was only 50 ms.

### Results

#### Reaction times

**Fig. 3:**
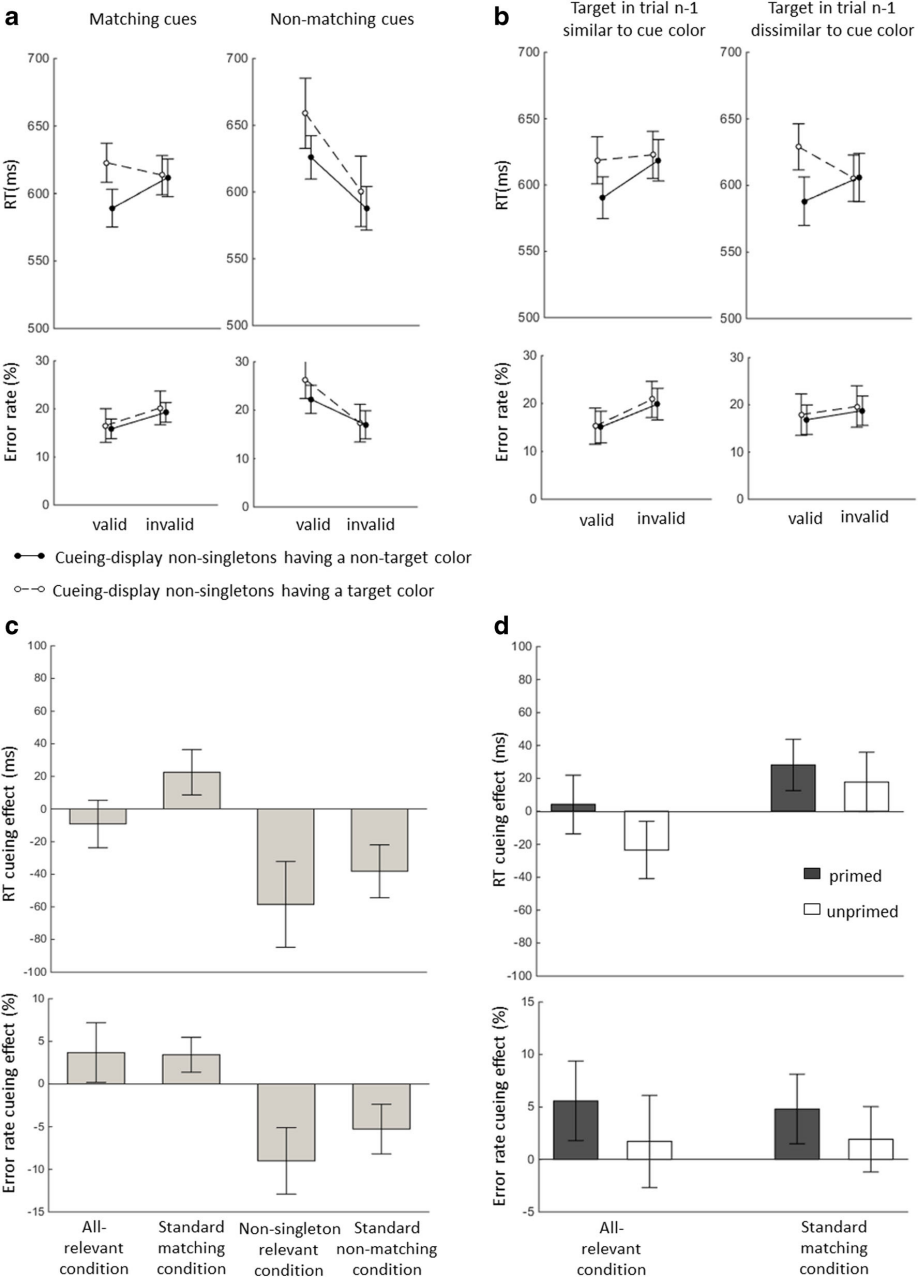
Data from Experiment 2. (A) Overall means of the reaction times (RTs) and error rates for the top-down matching and nonmatching cues. (B) Mean RTs and error rates for top-down-matching cues when the target in trial *n*−1 was similar to the cue in trial *n* (i.e., when the cue was primed) and when the color of the target in trial *n*−1 was dissimilar to the cue color in trial *n* (i.e., when the cue was not primed). Error bars represent the 95% confidence interval (CIs) of the invalid minus valid differences in the corresponding conditions. (C) Mean cueing effects (difference between mean RTs and error rates in the invalid and valid conditions) with different cueing displays. The upper panel corresponds to the cueing effects on RTs, and the lower panel corresponds to the cueing effects on error rates. The error bars depict the 95% CIs. (D) Mean cueing effects and CIs for RTs and error rates in the standard matching cue condition and in the all-relevant condition for trials in which the cue colors were primed and unprimed by the target color in trial *n*−1

The data for this experiment are shown in Fig. 3. The results of the ANOVA with the variables validity, cue match, and nonsingleton color on the trimmed means (2.2% of trials were excluded) of the correct RTs can be found in Table 3. Most importantly, we again found a two-way interaction between validity and nonsingleton color, *F*(1, 17) = 12.79, *p* < .01, *η*_p_^2^ = .43. Follow-up *t* tests revealed a cueing effect in the standard matching condition, 23 ms, *t*(17) = 3.41, *p <* .01, BF_10_ = 13.23, but not with the all-relevant cueing displays, − 9 ms, *t*(17) = 1.34, *p* = .20, BF_10_ = 0.52. With nonmatching cues, the cueing effects reversed significantly, − 59 and − 38 ms, *ps* < .01, BFs_10_ > 143.84.

**Table 3:** ANOVA results for Experiment 2, with the variables validity, cue match, and nonsingleton color

Source	*df*	*df* (error)	ANOVA on Reaction Times			ANOVA on Error Rates		
			*F*	*p*	*η*_p_^2^	*F*	*p*	*η*_p_^2^
Validity	1	17	11.25	<.01^**^	.40	9.30	<.01^**^	.35
Cue match	1	17	11.89	<.01^**^	.41	12.24	<.01^**^	.42
Nonsingleton color	1	17	15.44	<.01^**^	.48	4.41	.05	.21
Validity × Cue match	1	17	38.46	<.01^**^	.69	30.80	<.01^**^	.64
Validity × Nonsingleton color	1	17	12.79	<.01^**^	.43	1.68	.21	.09
Cue match × Nonsingleton color	1	17	1.09	.31	.06	1.98	.18	.10
Validity × Cue match × Nonsingleton color	1	17	1.59	.22	.09	1.86	.19	.10

^**^p < .01

#### Error rates

The results of the ANOVA on the percentages of the error rates are also in Table 3. Follow-up *t* tests revealed a cueing effect with the all-relevant cueing displays, 4%, *t*(17) = 2.21, *p* < .05, BF_10_ = 1.69, and with the standard matching cue conditions, 3%, *t*(17) = 3.52, *p* < .01, BF_10_ = 16.45. With nonmatching cues, the cueing effects reversed (*p*s < .01, BFs_10_ > 28.47).

### Intertrial priming

We investigated intertrial priming as in Experiment 1. Table 4 shows the results of an ANOVA with the variables validity, target *n*−1, and nonsingleton color. At variance with the bottom-up view, we observed a positive cueing effect with all-relevant cueing displays neither when the cue was primed, 4 ms, *t*(17) = 0.49, *p* = .63, BF_10_ = 0.27, nor when it was unprimed, − 24 ms, *t*(17) = 2.86, *p* < .05, BF_10_ = 4.92. With standard matching cueing displays, in contrast, we found a marginally significant cueing effect when the cue was unprimed, 18 ms, *t*(17) = 2.07, *p* = .05, BF_10_ = 1.36.

**Table 4:** Results of ANOVAs of intertrial priming effects in Experiment 2, with the variables validity, target *n*−1, and nonsingleton color

Source	*df*	*df* (error)	ANOVA on Reaction Times			ANOVA on Error Rates		
			*F*	*p*	*η*_p_^2^	*F*	*p*	*η*_p_^2^
Validity	1	17	1.69	.21	.09	13.00	<.01^**^	.43
Target *n*−1	1	17	3.51	.08	.17	0.44	.52	.03
Nonsingleton color	1	17	12.47	<.01^**^	.42	1.03	.32	.06
Validity × Target *n*−1	1	17	8.05	.011^*^	.32	3.20	.09	.16
Validity × Nonsingleton color	1	17	12.85	<.01^**^	.43	0.02	.88	.00
Target *n*−1 × Nonsingleton color	1	17	0.37	.55	.02	0.05	.83	.00
Validity × Target *n*−1 × Nonsingleton color	1	17	2.16	.16	.11	0.17	.69	.01

^*^p < .05, ^**^p < .01

For error rates, *t* tests revealed cueing effects when the cue was primed in all-relevant cueing displays, 6%, *t*(17) = 3.12, *p* < .01, BF_10_ = 7.79, and in standard matching cueing displays, 5%, *t*(17) = 3.06, *p* < .01, BF_10_ = 7.09. There were no cueing effects when the cue was not primed: 2%, *t*(17) = 0.83, *p* = .42, BF_10_ = 0.33, in all-relevant conditions, and 2%, *t*(17) = 1.31, *p* = .21, BF_10_ = 0.51, with standard matching cueing displays.

## Discussion

We investigated in a new condition of the contingent-capture protocol (Folk et al., 1992) to which extent the attention capturing potential of singleton cues exceeds that of nonsingletons when both singletons and nonsingletons had a searched-for target feature. Participants searched for two target colors, so that it was possible to create all-relevant cueing displays with a top-down matching singleton cue presented amidst three nonsingletons having the other target color. Hence, all objects in these cueing displays had a possible target color. If the singleton cue attracted attention in a bottom-up way, a regular cueing effect should have emerged in this all-relevant condition because disengagement from the cue based on a mismatch between cue and target features (Belopolsky et al., 2010; Theeuwes, 2010) was ruled out. If attentional capture was contingent on top-down search settings, no cueing effects should have emerged with all-relevant cueing displays because all display locations had objects with searched-for target features.

The results of both experiments supported the top-down contingent-capture hypothesis, showing that the cueing effects were strongly reduced (and almost absent) for the all-relevant cueing displays as compared to the standard matching cue conditions. It is true that for error rates there were residual cueing effects with all-relevant cueing displays. However, as the intertrial analysis showed, these effects were only present when the cues’ color in a trial *n* had been primed by the target color in trial *n*−1. When the cue was unprimed, the cueing effect with all-relevant cueing displays was absent (and even tended to be reversed).

The patterns of results were similar in both experiments, even though the SOA was reduced from 150 ms in Experiment 1 to 50 ms in Experiment 2. This is important, because one could argue that the presence of target features in the nonsingletons of the all-relevant cueing display might have fostered disengagement from the cue. However, in light of Experiment 2 even this possibility is not likely, because the SOA was most likely too short for rapid disengagement (cf. Kim & Cave, 1999; see also Chen & Mordkoff, 2007).

### Reversal of cueing effects with nonmatching cues

The reversal of the cueing effect in the 50-ms SOA in conditions with nonmatching cues was also remarkable. The reversal of cueing effects is sometimes observed in contingent-capture experiments and has previously been attributed to either object updating in working memory (Carmel & Lamy, 2014) or other attentional mechanisms, such as the suppression of task-irrelevant features (for a more detailed review, see Schoeberl, Ditye, & Ansorge, 2018; see also Gaspelin & Luck, 2018). In any case, it is clear that the reversal of the cueing effects with nonmatching cues is the opposite of what one would expect if singleton cues captured attention in a bottom-up way. It is possible, however, that the reversal of the cueing effect reflected a form of salience-based suppression (Gaspelin & Luck, 2018).

### Conclusions

Most importantly, the present study suggests that the attention-capturing potential of salient singleton cues is no greater than that of nonsingletons when both singleton cues and nonsingletons are presented in top-down matching colors. More specifically, the present results rule out the possibility that rapid disengagement from the cue’s location, which is based on mismatch between the cue’s features and the searched-for target features, is responsible for the lack of cueing effects in standard nonmatching cue conditions in the contingent-capture protocol. The present results therefore favor models that assume that attentional capture is already under top-down control at early stages of processing (Folk et al., 1992).

Yet, salience undeniably has a strong impact on attentional capture under certain conditions (e.g., Theeuwes, 1992). The human visual system may be remarkably flexible regarding the way that salience is processed and captures attention. Under certain conditions, people might use a salience detection mode (cf. Bacon & Egeth, 1994). That is, people might allow for salient stimuli to be processed in an efficient way. However, under other conditions it is possible to overcome the impact of salience almost completely—for instance, when people have to search for a specific feature target, as was the case in the present study.

## Footnotes

1 Notice that cueing effects should not be considered pure measures of attentional capture, because rejection time after initial capture can contribute to the net cueing effects (e.g., DiQuattro & Geng, 2017; Fukuda & Vogel, 2011; Gaspelin, Ruthruff, & Lien, 2016).

2 Several studies have demonstrated that participants can adopt search settings for at least two colors simultaneously (e.g., Folk & Remington, 2008; Irons, Folk, & Remington, 2012).

3 The onset-search condition in the classical paradigm (Folk et al., 1992) is similar to this all-relevant condition, because onsets appeared at all display locations when color cues were presented. However, search settings for the target’s color play a role when participants search for abrupt onsets (Goller, Ditye, & Ansorge, 2016). It is hence possible that disengagement might occur during onset search because of a mismatch between the cue and the target color.
